# Case Report: Whispers of the serpent: exploring uncommon imaging features in primary hepatic malignant mesothelioma

**DOI:** 10.3389/fmed.2025.1713971

**Published:** 2025-11-12

**Authors:** Guoan Li, Shengqian Hong, Tao He, Jianbo Xu

**Affiliations:** The Affiliated Huaian No.1 People's Hospital of Nanjing Medical University, Huai'an, China

**Keywords:** primary hepatic malignant mesothelioma, serpiginous peripheral enhancement, contrast-enhanced CT/MRI, immunohistochemistry, hepatic resection

## Abstract

Primary hepatic malignant mesothelioma (PHMM) is an uncommon and aggressive neoplasm with vague clinical and radiological features, posing challenges for preoperative diagnosis. In our case, a lobulated hepatic mass demonstrated a serpiginous peripheral enhancement pattern on contrast-enhanced CT and MRI. This uncommon imaging manifestation has been sporadically documented in previous reports. By consolidating these findings, our report emphasizes serpiginous peripheral enhancement as a potential diagnostic clue for PHMM. Recognition of this pattern may aid earlier detection, improve differential diagnosis, and guide timely surgical decision-making in affected patients.

## Introduction

Malignant mesothelioma is an uncommon yet highly aggressive neoplasm arising from mesothelial cells. It most frequently affects the pleura (approximately 70% of cases) and the peritoneum (about 20%), whereas occurrences in the pericardium and tunica vaginalis are considerably rarer (1). PHMM is extremely rare, with fewer than 20 cases reported globally. Due to its rarity, PHMM lacks established clinical characteristics. Most patients present with nonspecific abdominal discomfort, and laboratory and imaging examinations also lack specificity. As such, preoperative diagnosis is difficult, and final confirmation depends on pathological and immunohistochemical analysis.

Here, we describe a case of PHMM treated at our institution and provide a brief literature review to highlight its diagnostic challenges and therapeutic strategies.

## Case data

A 66-year-old male farmer was admitted to our hospital with a recurrence of right upper abdominal discomfort over the past week. He had no history of alcohol abuse, chronic hepatitis, or cirrhosis, denied any asbestos exposure, and reported no other comorbidities. On physical examination, mild right upper quadrant tenderness was noted without hepatomegaly, splenomegaly, or ascites. The patient initially visited an external hospital, where he was misdiagnosed with a liver abscess and underwent a needle biopsy. Despite the poor drainage and the biopsy finding of only liver fibrosis, the patient was advised to continue observation at home while completing a course of oral antibiotics. Three months later, he revisited our hospital for re-evaluation, during which a CT showed that the hepatic mass had not decreased or resolved.

## Treatment and diagnosis

**Figure 1:**
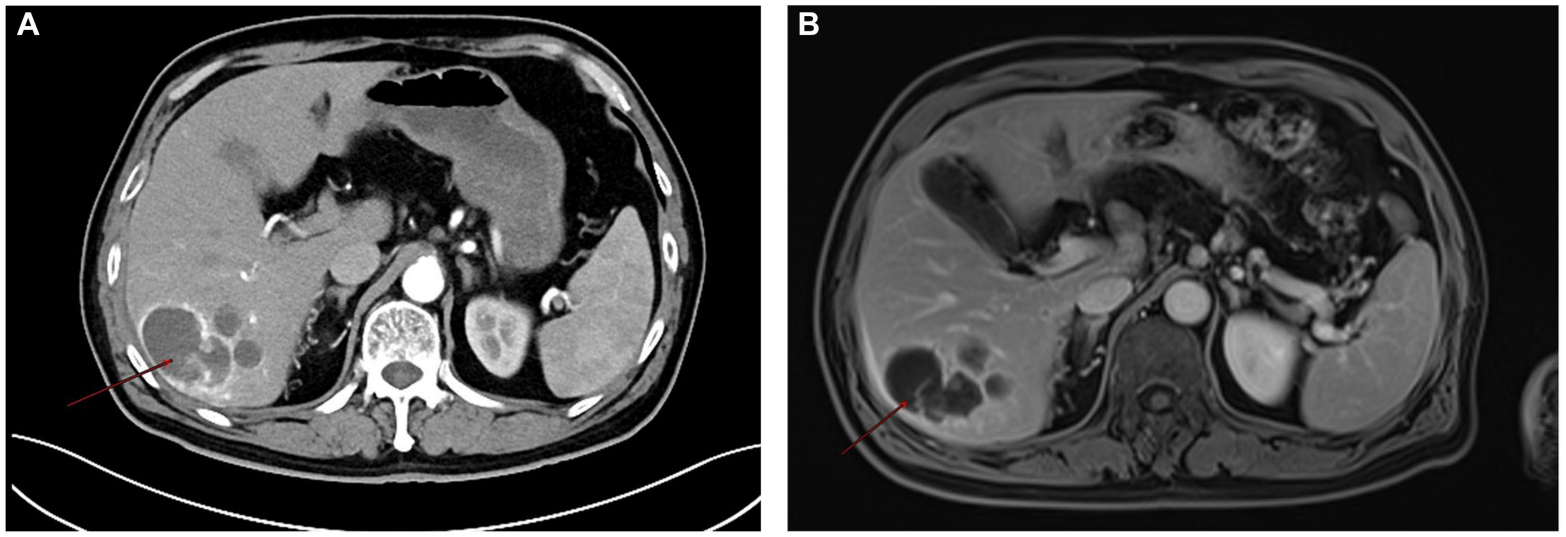
Contrast-enhanced CT **(A)** showing a hypodense lesion in segments VI-VII of the liver, measuring approximately 5.8 cm. The lesion demonstrates mild heterogeneous enhancement in the arterial phase with a serpiginous, serpentine distribution of enhancement along the peripheral region. MRI **(B)** also exhibits heterogeneous enhancement after gadolinium injection, with a serpiginous, serpentine distribution of enhancement observed along the peripheral region.

**Figure 2:**
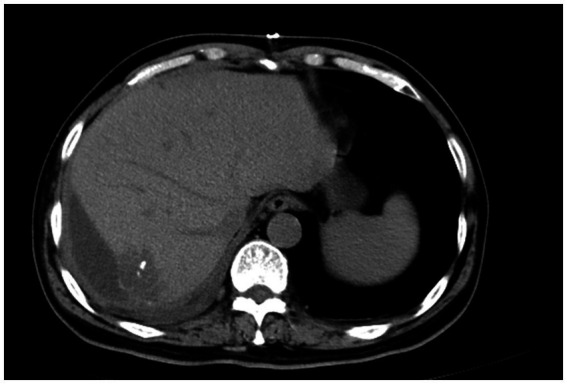
Postoperative CT scan showing complete tumor resection with minimal perihepatic fluid accumulation.

Routine laboratory tests, including complete blood count, liver function, and renal function, were within normal limits. Serum tumor markers including CA19-9, CEA, and AFP were negative. Contrast-enhanced CT: A hypodense lesion in segments VI-VII of the liver measuring approximately 5.8 cm. The lesion showed mild heterogeneous enhancement in the arterial phase and partial washout in the portal venous phase, with a serpiginous distribution of enhancement along the peripheral region (Figure 1A). MRI: A 5.6-cm lobulated lesion in the liver, T1 hypointense and T2 hyperintense, with heterogeneous enhancement after gadolinium injection. Diffusion-weighted imaging showed restricted diffusion. A serpiginous distribution of enhancement was observed along the peripheral region (Figure 1B). Due to the patient’s prior needle biopsy and drainage procedure three months ago, which showed poor drainage and liver fibrosis without resolution of the mass, and the fact that the lesion had not decreased in size after three months of follow-up, the lesion remained indeterminate. Given the indeterminate nature of the lesion and the inability to exclude malignancy based on preoperative imaging, surgical resection was performed to ensure oncological safety. This decision was also in line with the patient’s request for definitive treatment. A liver resection was performed with a margin of at least 1 cm from the tumor to ensure the complete removal of potentially involved tissue while preserving sufficient residual hepatic tissue to maintain adequate liver function and achieve clear surgical margins. During surgery, the tumor, located on the liver surface near the liver-renal interface, posed a significant surgical challenge. Its delicate surrounding tissues made dissection difficult and increased the risk of bleeding. Nevertheless, the team successfully achieved complete tumor resection with negative margins. Intraoperative frozen section analysis confirmed a malignant hepatic tumor with cellular atypia. The patient’s postoperative recovery was uneventful: they began oral fluids on postoperative day (POD) 1, mobilized by POD 3, and was discharged in stable condition on POD 13 following an excellent recovery (Figure 2). This recovery trajectory is consistent with the patient’s good general health and the absence of major postoperative complications. Taking into account the rarity of the tumor, this approach was chosen to ensure oncological safety while also minimizing risks to liver function.

**Figure 3:**
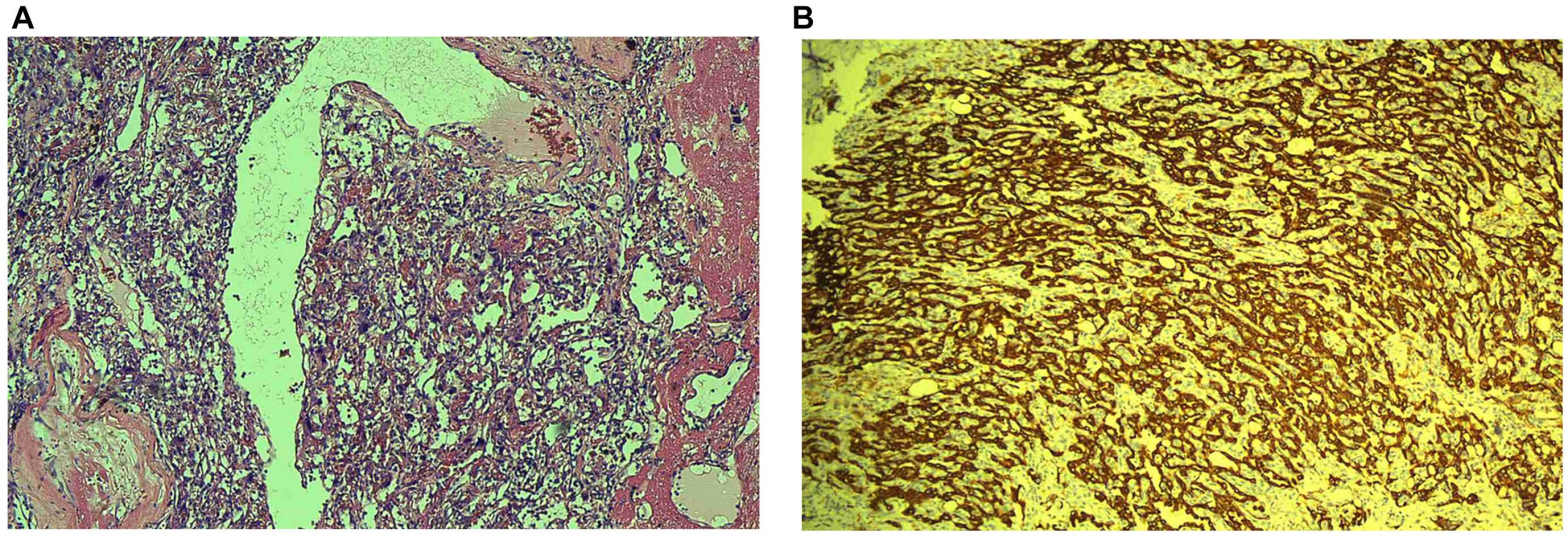
Histopathological **(A)** image of primary hepatic malignant mesothelioma (PHMM) stained with hematoxylin and eosin (HE). The tumor exhibits epithelioid cells arranged in sheets and nests, with nuclear pleomorphism, frequent mitotic figures, and invasive growth into the surrounding liver tissue, characteristic of malignant mesothelioma. Immunohistochemistry **(B)** showing positive staining for Calretinin (CR) and WT-1, indicative of mesothelial origin. The tumor cells also show strong D2-40 (Podoplanin) positivity, supporting the diagnosis of PHMM.

Postoperative histopathological analysis revealed the tumor was composed of epithelioid cells arranged in nests and sheets, with nuclear pleomorphism, frequent mitotic figures, and focal necrosis (Figure 3A). Immunohistochemistry showed tumor cells were positive for CK (3+), WT-1 (1+), CK5/6 (1+), D2-40 (2+), CR (3+) and negative for Hepatocyte, CD31, CD34, SMA, HMB45 (Figure 3B). These findings were consistent with PHMM.

**Figure 4:**
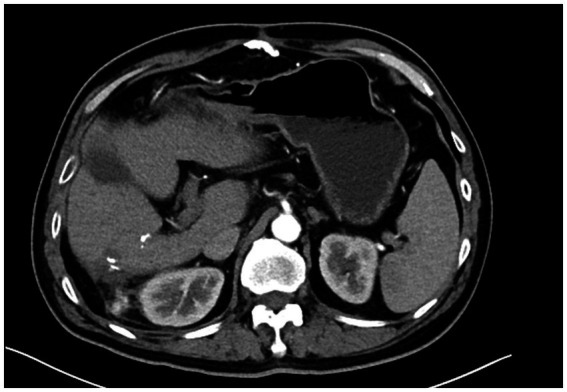
Postoperative follow-up CT scan after three months showing no signs of recurrence, with stable liver and surrounding structures.

**Figure 5:**
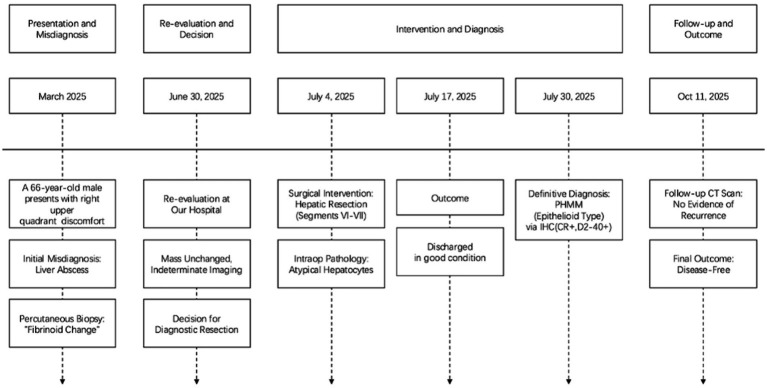
Clinical timeline summarizing key events of presentation, diagnosis, treatment, and follow-up, aligned with the CARE guidelines.

The patient was scheduled for the first follow-up visit three months after discharge. During this follow-up, contrast-enhanced CT imaging was performed, which showed no signs of tumor recurrence (Figure 4). Given the rarity of PHMM and the lack of well-established treatment guidelines, it was decided to adopt a more conservative approach in terms of follow-up. As such, the patient will continue with a surveillance plan similar to that of other malignant liver tumors, with CT imaging scheduled every three months for the first two years post-surgery. The clinical timeline figure following the CARE guidelines has been added as Figure 5.

## Discussion

PHMM is a rare mesothelial malignancy. Reports of PHMM in the past 5 years are shown in Table 1. Unlike pleural mesothelioma, asbestos exposure is not clearly associated with hepatic mesothelioma (2). Its pathogenesis remains uncertain, with hypotheses including mesothelial cell rests in Glisson’s capsule undergoing malignant transformation (3). Owing to its nonspecific radiological manifestations, the preoperative diagnosis of PHMM remains challenging. In the present case, contrast-enhanced CT revealed a serpiginous peripheral enhancement pattern, an imaging feature that is unusual in common hepatic tumors but has been sporadically described in previously reported PHMM cases. Serter et al. (4), Leonardou et al. (5), and Jia et al. (6) have clearly revealed a serpiginous peripheral enhancement pattern in their studies, which aligns with the imaging findings observed in our case (4–6). We also observed a serpiginous peripheral structure on the enhanced CT images provided in the study by Dong et al. (7). However, this feature was not explicitly emphasized by the authors. It is important to note, however, that serpiginous peripheral enhancement is not unique to PHMM. Similar findings may be encountered in other hepatic lesions: hepatic hemangiomas typically show peripheral nodular enhancement with centripetal fill-in, a dynamic pattern not observed in PHMM (8). On MRI, hemangiomas often demonstrate the characteristic “light-bulb sign,” appearing markedly hyperintense on T2-weighted images, which further distinguishes them from PHMM. Focal nodular hyperplasia (FNH) is often associated with a central scar that enhances in a radiating fashion on delayed phases (9). Intrahepatic cholangiocarcinoma (ICC) usually demonstrates progressive delayed enhancement and is frequently accompanied by bile duct dilatation and elevated tumor markers such as CA19-9 or CEA (10), whereas in our case, all tumor markers were within normal limits.

**Table 1:** Overview of PHMM cases in the last 5 years.

Case source (First author)	Year	Age/Sex	Tumor size (cm)	Asbestos Exposure	Histologic Type	Imaging manifestations	Treatment	Outcome (Follow-up)
Ghimire (2)	2020	70 /M	8.0	Yes	Epithelioid	No specific enhancement pattern detailed.	Surgery + Adjuvant chemotherapy	No recurrence (15 months)
Pernthaler (12)	2023	48 /F	Not reported	Not reported	Epithelioid	Large inhomogeneous tumor; pathological FDG uptake at margins with central hypometabolism (necrosis).	Chemotherapy + Surgery	Not reported
Wei (13)	2023	69 /M	13.5	Not Reported	Sarcomatoid	Mixed signal mass with blurred borders; enhancement at the edge and septa.	Not reported	Not reported
Mehta (14)	2023	72 / M	13.6	No	Sarcomatoid	Large cystic mass with heterogeneous components.	Palliative Chemotherapy	Died (within months)
Jia (Case 1) (6)	2024	53 / F	1.7	No	Epithelioid	Solid, heterogeneous soft tissue mass with irregular margins and significant marginal enhancement in arterial phase.	Supportive care	Died (20 days)
Jia (Case 2) (6)	2024	54 /F	9.9	No	Epithelioid		Right hemihepatectomy	Died, with recurrence (9 yrs. 8 mo)
Jia (Case 3) (6)	2024	50 /F	14.0	No	Epithelioid		Left Hemihepatectomy	Alive, no recurrence (14 mo)
Jiang min (15)	2024	65 /F	8.5	No	Biphasic	Irregular mass with internal septations; heterogeneous enhancement.	Chemotherapy	No progression (8 months)
Li (This case)	2025	66 /M	5.8	No	Epithelioid	Serpiginous enhancement along the peripheral region.	Surgical resection	No recurrence(3 mo)

PHMM, primary hepatic malignant mesothelioma; M, male; F, female.

Epithelioid, sarcomatoid, and biphasic are the three types of malignant mesothelioma, with epithelioid being the most common (2). Our case also presents as the epithelioid type. The positive results for immunohistochemical markers Calretinin (CR), WT-1, and D2-40 (Podoplanin), along with a negative result for CD34, support the diagnosis of primary hepatic mesothelioma (11). Due to its rarity, there is no standardized treatment for this condition. In previous studies, most early-stage patients underwent surgical treatment and achieved favorable prognoses. Therefore, surgical resection was also chosen for this patient, with the hope of a positive outcome.

The distinctive contribution of this report lies in consolidating the serpiginous peripheral enhancement pattern as a recurrent imaging feature of PHMM. While individual cases have sporadically described this manifestation, our report not only documents the finding in detail but also contextualizes it within the existing literature. By emphasizing this uncommon yet reproducible feature, the present case adds to the limited pool of evidence that may assist radiologists and clinicians in suspecting PHMM earlier and differentiating it from other hepatic tumors.

## Conclusion

While the imaging characteristics of PHMM are not yet fully defined, the presence of serpiginous peripheral enhancement warrants attention. With the accumulation of more case reports, this finding may contribute to earlier recognition and more accurate differential diagnosis of PHMM.

## Data Availability

The original contributions presented in the study are included in the article/supplementary material, further inquiries can be directed to the corresponding author/s.
